# The Mechanism of Ultrasonic Vibration on Grain Refining and Degassing in GTA Spot Welding of Copper Joints

**DOI:** 10.3390/ma11050737

**Published:** 2018-05-07

**Authors:** Salih Al-Ezzi, Gaofeng Quan, Adil Elrayah

**Affiliations:** 1 Key Laboratory of Advanced Technologies of Materials, Ministry of Education, School of Material Science and Engineering, Southwest Jiaotong University, Chengdu 610031, China; salihlink@yahoo.com (S.A.-E.); adil.karary@yahoo.com (A.E.); 2 Engineering Technical College-Baghdad, Middle Technical University, Baghdad 10074, Iraq

**Keywords:** GTA spot welding, copper, grain refinement, tensile shear strength, degassing, corrosion

## Abstract

This paper examines the effect of ultrasonic vibration (USV) on grain size and interrupted porosity in Gas Tungsten Arc (GTA) spot-welded copper. Grain size was refined by perpendicularly attaching a transducer to the welded sheet and applying USV to the weld pool for a short time (0, 2, 4, and 6 s) in addition improvements to the degassing process. Results illustrate a significant reduction of grain size (57%). Notably, USV provided interaction between reformations (fragmentation) and provided nucleation points (detaching particles from the fusion line) for grains in the nugget zone and the elimination of porosity in the nugget zone. The GTA spot welding process, in conjunction with USV, demonstrated an improvement in the corrosion potential for a copper spot-welded joint in comparison to the joint welded without assistance of USV. Finally, welding of copper by GTA spot welding in conjunction with ultrasound for 2 s presented significant mechanical properties.

## 1. Introduction

The market requirements of copper grow annually [1], especially for electrical applications. Additionally, at present, the high-strength connection of materials, including aluminum, magnesium, copper, and titanium alloys, have been realized [2]. Moreover, as a result of the increase of copper auxiliary motors and copper electric connections of devices in hybrid cars, weight reduction is a highly relevant topic in car body design [3]. The small, narrow thickness of copper sheets present several limitations in welding processes: (1) there is little back support to complete the friction stir welding process [4]; (2) copper has a low absorption of laser beams with infrared wavelength in laser welding processes [5]; and (3) the use of filler metal reduces the electrical conductivity of the copper joint [6]. Therefore, it is difficult to obtain a joint for thin copper sheets with high electrical conductivity. On the other hand, the presence of porosity in the nugget zone (NZ) of a pure copper-welded joint reduces the electrical conductivity [7] , in which porosity at NZ is caused by a high diffusivity of hydrogen in the weld pool during Gas Tungsten Arc Welding process (GTAW) [8]. Although the effect of ultrasonic vibration (USV) on grain refinement and hydrogen removal from non-ferrous light metal alloys was examined [9,10,11], research on nugget degassing for heavy non-ferrous metal melts such as copper is still lacking. For gas metal arc welding, the lengthy welding time may reduce porosity [12] and refine the grains. Additionally, it has been reported that a disruption of columnar grains in the nugget area increases the tensile strength of the copper welded joint [4]. Recently, ultrasound was integrated with the GTAW process to improve the penetration, material mixing, reduction of porosity, weld quality, arc pressure, and grain size [13].

However, the presence of porosity at the copper NZ has been noticed in many fusion welding studies, and the grain size for weldments of pure copper metal are more significant than weldments of copper alloys. In this study, welding pure copper sheets by the GTA spot welding process is proposed and tested via experiment in which a transducer was attached underneath the lower plate. USV was initiated and interacted directly after the end of the main arc application. Various vibration times were assessed (0, 2, 4, and 6 s). Additionally, tensile shear tests, microstructure, electrical conductivity, and corrosion resistance were investigated. Significant attention was paid to the mechanism of enhancement and its effect on the investigated properties.

## 2. Materials and Methods

**Figure 1:**
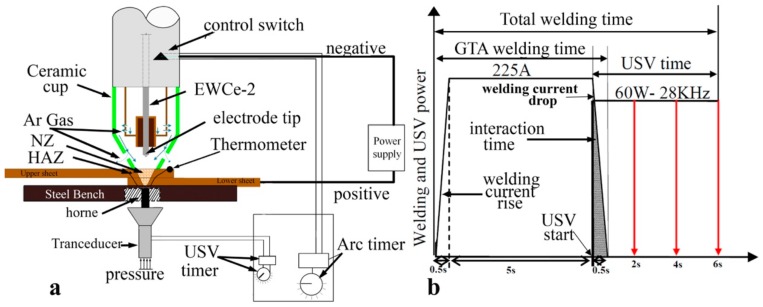
(**a**) Schematic diagram of GTA spot welding assisted with ultrasound; and (**b**) the power application cycle of the welding current and ultrasonic power time.

Pure copper, 99.97%, with dimensions 100 mm× 25.4 mm× 1.2 mm was welded with GTA spot welding to achieve a fluxless and fillerless joint (Figure 1a). A transducer at 28 KHz and 60 watts was attached below the steel bench with adequate pressure. The horn of the transducer was inserted through a hole 12 mm in diameter in the steel bench to be at 5 mm from the copper spot-welded joint. It was activated during the solidification time of the copper weld pool for 0, 2, 4, and 6 s. The interaction of ultrasonic vibration power with the welding time during the drop of arc welding current with 0.5 s is shown in Figure 1b. Additional welding parameters, such as electrode type, electrode diameter, electrode tip angle, welding current, arc time, gas type, and gas flow rate, were kept constant as follows: an EWCe-2 electrode, 3.2 mm, 120°, 225A, DCEN (Direct Current Electrode Negative), 5 s, argon, and 12 L/min, respectively. The process was semi-automated, in which the welding machine and ultrasonic device were connected entirely to an electric control circuit. The strength requirement for spot welds are normally specified in load unit/weld [12], and tensile-shear testing is a common test for investigating the mechanical performance of spot welds under static conditions [14,15,16]; accordingly, three welded joints were tested in an MTS-CMT 5105 universal testing machine (SANS, Shenzhen, China) at room temperature to evaluate the joint strength for each condition. The crosshead speed was 1 mm/min. Zeiss OM and Scanning electron microscope (SEM, Quanta FEG250, Hillsboro, OR, USA) equipped with X-ray diffraction Spectroscopy (EDS, PANalytical B.V., Almelo, Holland) were used to characterize the macro- and microstructure of the joint for the upper portion and cross-section of the nugget zone (NZ) and heat-affected zone (HAZ). The samples were ground and polished then etched with 5 g FeCl_3_, 5 mL HCl, and a few drops of HNO_3_ and 100 mL water for a few seconds at room temperature. The temperature history of the HAZ was recorded at 12.5 mm from the center of NZ (3 mm from the ceramic cup) by a four-input data logger-type DT-3891G digital thermometer (CEM-instruments, Shenzhen, China). Samples with dimensions of 30 mm × 25 mm × 2.4 mm were cut from a lapped joint then ground and polished for electrical conductivity testing with a D60K-E eddy current electrical conductivity measurement system (Suzhou Desisen Electronic Technology Co., LTD, Suzhou, China). Corrosion potential and current at the cross-section of the welded joint were recorded on a Zahner IM6e electrochemical workstation (ZAHNER-elektrik GmbH & Co., KG, Germany) using a solution of 3.5 wt % NaCl. The standard calomel electrode and Pt were used as a reference electrode and auxiliary electrode, respectively. The polarization curve was recorded by means of potential-dynamic scanning with a scan rate of 1 mV/s and analyzed by Tafel straight-line extrapolation.

## 3. Results

In this study, an ultrasonic transducer with a frequency 28 KHz and 60 W power was attached to copper welded plates proposed to refine and degas grains in the nugget zone.

### 3.1. The Microstructure of GTA Spot-Welded Copper Joints

At temperatures above approximately 450 °C, absorbed hydrogen in molten copper reacts with the inclusions to form steam in the microstructure [17]:

Cu2O + 2H → 2Cu + H2O (steam)

**Figure 2:**
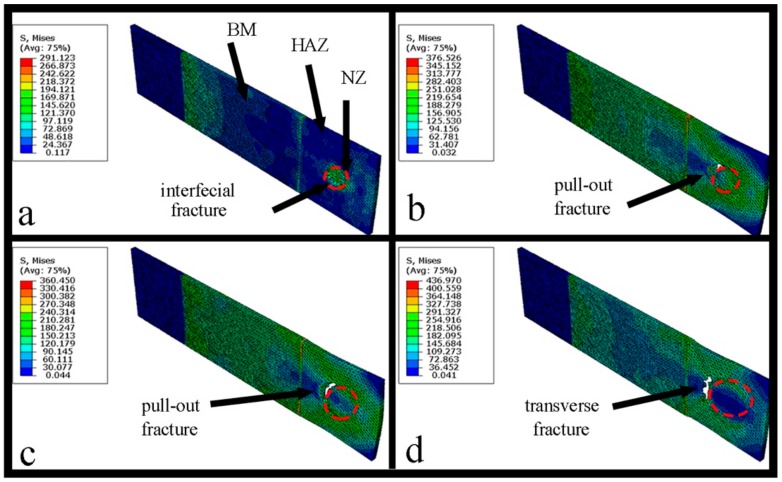
The simulated tensile-shear test of a copper spot-welded joint with a nugget diameter of (**a**) 5 mm; (**b**) 7.5 mm; (**c**) 10 mm; (**d**) 12.5 mm.

**Figure 3:**
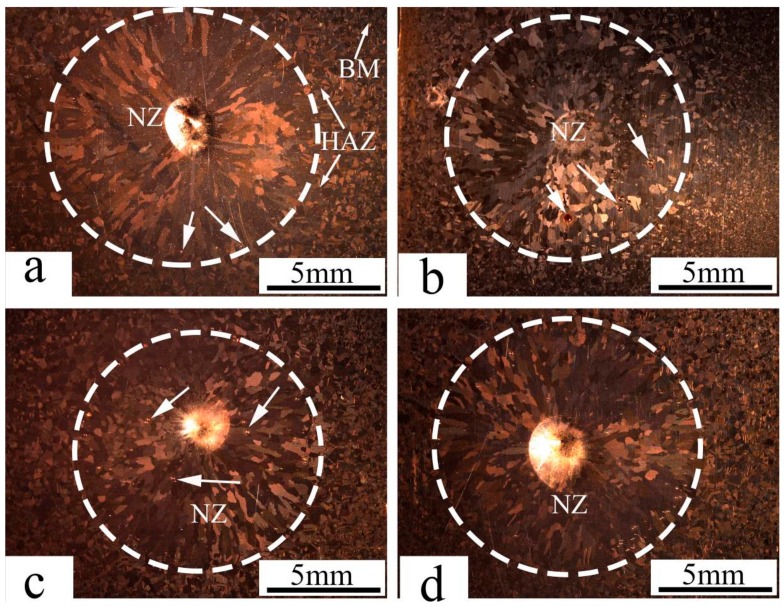
The macrostructure of GTA spot-welded copper joints with the assistance of ultrasonic vibration (USV) for (**a**) 0 s (without USV); (**b**) 2 s ; (**c**) 4 s; and (**d**) 6 s.

The macroporosities can be interrupted at the fusion line beside the NZ, which represent a critical reason to initiate a fracture. Half of the GTA spot-welded copper joints were simulated with Abaqus CAE software which is symmetrical at two sides of the nugget. The simulation consisted of the application of an axial tensile load at the edge of the sample and an encastre boundary condition at the NZ contact surface. The fracture of the simulated spot-welded joint with nugget sizes of 5, 7.5, 10, 12.5 mm are shown in Figure 2a–d, respectively. The result implies that the fracture location behavior changed from an interfacial fracture to pullout with a corresponding increase of the nugget diameter from 5 to 7.5 or 10 mm. With a further increase of the nugget diameter to 12.5 mm, the fracture behavior changed to a transverse fracture, initiated at the edge of the NZ and propagated to the HAZ. These results stood in good agreement with the results of Thibaut Huin investigation, in which it was determined that the failure location (interfacial, pull out, and transverse) depended on nugget size [18]. The entrapment of porosity which forms during gas welding processes is a factor of tensile-shear failure. The effect of USV on the macrostructure and porosity location is demonstrated in Figure 3. Figure 3a shows a GTA spot-welded copper joint without USV. Because the NZ cools rapidly, a sizeable columnar grain structure was observed in the NZ, followed by coarse grain structure in the HAZ and a fine grain structure in the base metal (BM), in addition to entrapped fine porosities within the fusion line of nugget zone.

**Figure 4:**
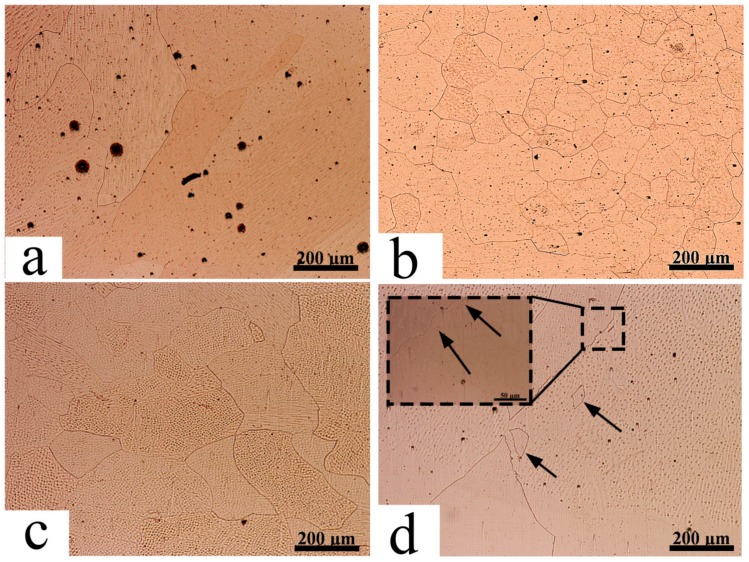
The evolution of the microstructure and porosity distribution in the nugget zone of GTA spot-welded copper joints welded without USV (**a**) and (with USV) for (**b**) 2 s; (**c**) 4 s; and (**d**) 6 s.

Macroporosities were increased on the surface with the presence of USV, as shown in Figure 3b,c. The increased porosity for USV for durations of 2 and 4 s were attributed to the cluster of microporosity. The increase of the USV duration to 6 s reduced the porosity, as demonstrated in Figure 3d in comparison to 2 and 4 s. USV moved the location of the pores to the center of joint. Figure 4a–c shows that USV activated the nucleation process and produced more equiaxed grains to be formed in the NZ; additionally, detached parts of grains from the fusion line moved inside the nugget zone, as shown in Figure 4d. The detached particles may have activated the nucleation of grains in the molten metal of the weld pool.

**Figure 5:**
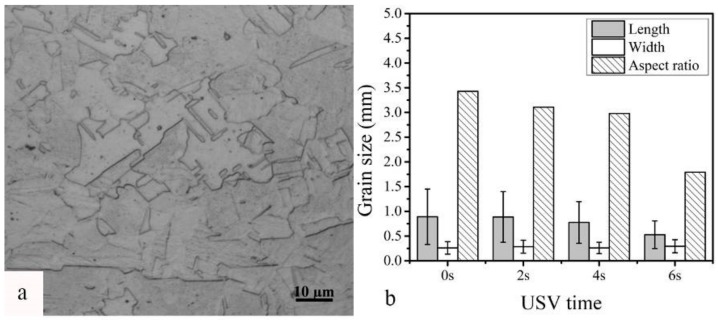
(**a**) The microstructure of the BM; and (**b**) the grain size and aspect ratio for GTA spot-welded copper joints with different USV times.

Grain size in the nugget zone of welds was measured following the procedures as described in ASTM E112-13—Standard Test Methods for determining average grain size. Figure 5a shows the fine grain size of the base metal (BM), and Figure 5b illustrates the reduction of the grain size with the increase of the USV time. The reduction of the grain size was related to the aspect ratio (grain length to width). The increase of the USV time to 6 s reduced the aspect ratio by 48% in comparison to the nugget zone welded without USV.

**Figure 6:**
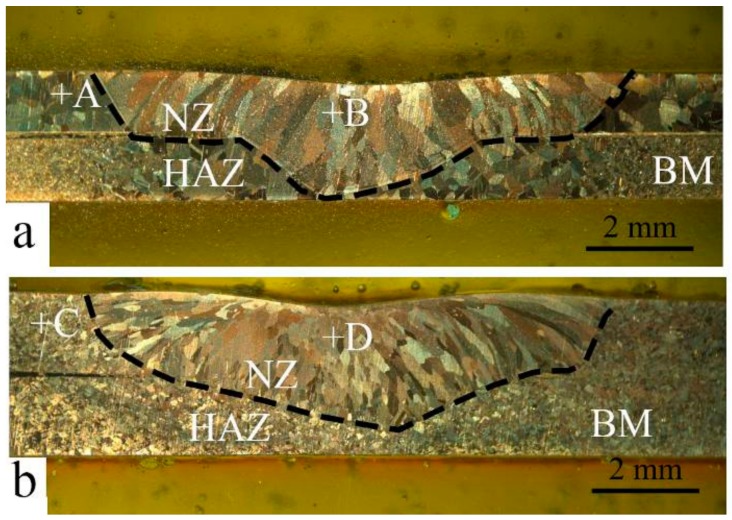
The cross-section macrostructure of a GTA spot-welded joint (**a**) without USV; and (**b**) with 6 s USV.

**Figure 7:**
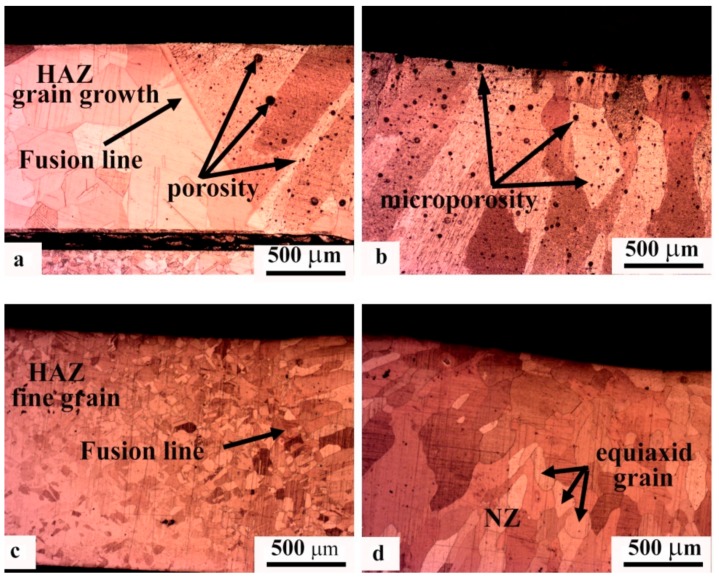
The cross-section microstructure of GTA spot-welded joints. (**a**,**b**) Locations labeled in Figure 4a for joints welded without USV; and (**c**,**d**) the locations labeled in Figure 4b for joints welded with USV assistance.

The investigation of the cross-section of welded joints also showed the reduction of grain size in the HAZ and NZ (Figure 6). For welded joints without USV, heat dissipated from the NZ increased the grain size in the HAZ; however, it did not show the same effect with the use of USV for 6 s, as shown in the macrostructure of joint. Figure 6 illustrates the cross-section macrostructure of joints with and without USV. The microstructure of welded joints revealed the presence of large grain sizes and microporosities in copper joints welded without USV, as shown in Figure 7a,b, in addition to grain growth in the HAZ. Figure 7c,d show copper joints of GTA spot welds assisted by USV for 6 s. The decrease in the grain size and the reduction of porosities is evident. The microstructure in Figure 7d reaffirms that microporosities were clustered and moved out of the NZ surface during USV.

**Figure 8:**
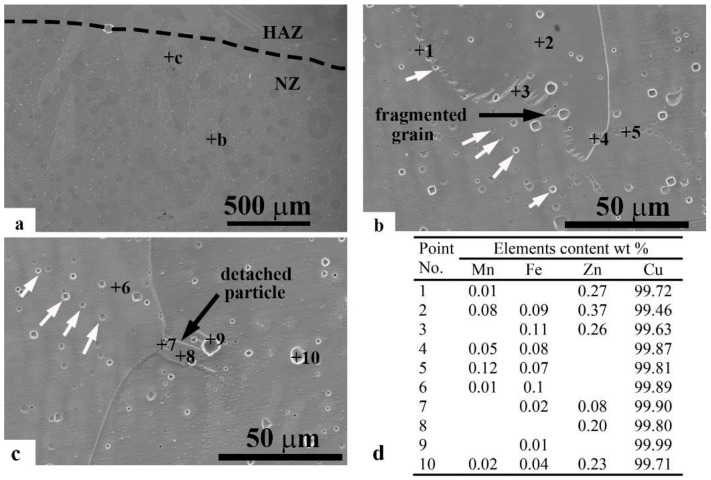
SEM observations of the GTA spot-welded joint assisted with 2 s USV (**a**) low magnification of the nugget surface; (**b**) high magnification for the fragmented grain labeled in a; (**c**) high magnification for the detached particle in the location labeled in a; and (**d**) EDS results of several characteristic regions labeled in b and c.

The detached particles and fragmented grains were investigated. Figure 8 illustrates the surface of a welded joint with 2 s USV. Having been affected by USV, the solidified fragmented grain moved into the weld pool toward the center. The chemical composition of the redistributed elements of copper showed the high-purity copper at the grain boundary as well as in grains where the GTA spot welding process was free of additives (filler and flux). The detached small particle (Figure 8c) showed a higher purity of copper than the solidified fragmented grain (Figure 8b). The uniform shapes (square and pyramid), indicated with white arrows, were attributed to dislocations within the grain, as shown in Figure 8b,c.

**Figure 9:**
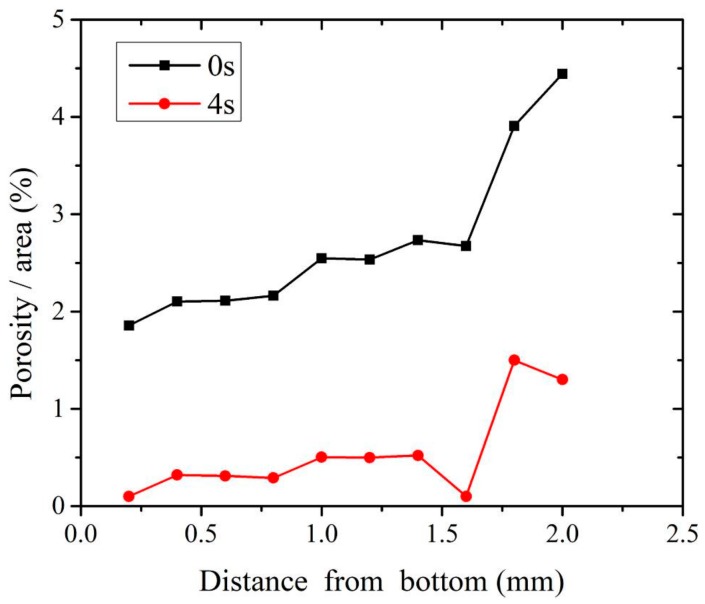
The distribution of porosity from bottom to top surfaces in GTA spot-welded joints with and without USV assistance.

The distribution of porosity through the nugget zone was investigated. Figure 9 illustrates the porosity area percent for cross-sections of welded joints with and without the assistance of USV. The degassing of porosity in the microstructure is evident. The increase of the porosity percent near the upper part represents movements of porosity from the lower plate to the surface of the weld pool during the solidification process. The activation of this movement with USV formed a joint with microporosity.

### 3.2. Strength of GTA Spot-Welded Copper Joints

**Figure 10:**
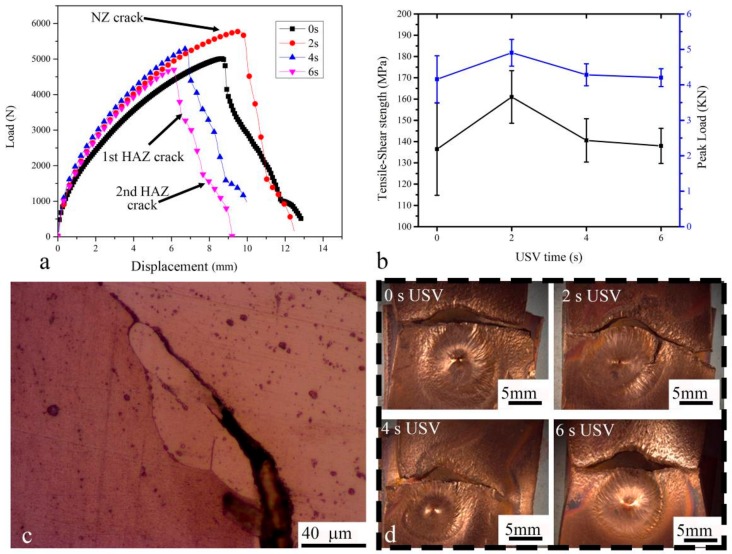
GTA spot-welded joints behavior with different times of USV (0, 2, 4, 6 s) tested in a tensile-shear test: (**a**) typical load-displacement curves; (**b**) tensile-shear strength and peak load vs. USV time; (**c**) the micro-solidification crack for a cross-sectioned sample welded with 6 s USV; and (**d**) the fracture location.

Figure 10a–c show typical welded joints with 0–6 s USV tested in a tensile-shear test. When USV increased from 0–4 s, the force increased, owing to degassing of the microporosity. As a result, the pores moved to the surface of joint instead of being interrupted in the NZ. This suggests that USV transferred to the NZ during the welding process which slightly improved the strength of the welded joint. On the other hand, the increase of USV to 6 s showed a decrease of peak load and strength, in which several micro-solidification cracks appeared in the fusion zone, as shown in Figure 10d. The load-displacement curves showed a similar behavior when the first crack started after the reduction of the area at the NZ, which was located at the upper plate, as shown in Figure 10c. The fracture transferred from the NZ to the HAZ, where the stress concentrated at that part of plate after separation of the NZ. The concentration of the load on the side of the HAZ may have increased the hardness of copper due to strain hardening; subsequently, a second crack appeared on another side of the HAZ. The transverse fracture indicated that the NZ and the interface had a higher strength than that of the base metal in the HAZ. Although the fracture completely occurred at the upper plate, the plate’s original area (25.4 × 1.2 mm^2^) was assumed to estimate the tensile-shear strength (Figure 10b). Finally, the USV for 2 and 4 s improved the tensile-shear strength, while the presence of micro-solidification cracks slightly reduced the strength of the welded joint with 6 s USV.

### 3.3. Fracture of GTA Spot-Welded Copper Joints

**Figure 11:**
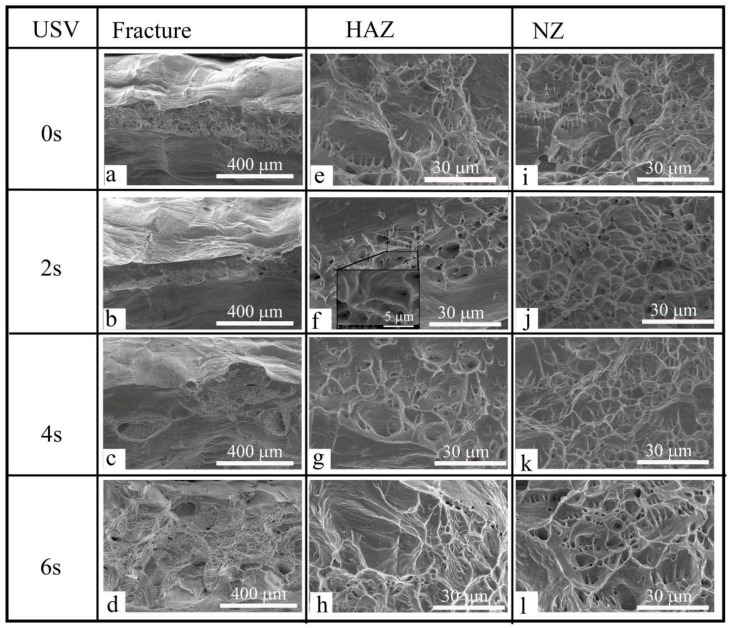
SEM images showing the fracture of copper GTA spot-welded joints with 0–6 s USV. (**a**–**d**) tensile-shear fracture surface; (**e**–**h**) HAZ fracture appearance; and (**i**–**l**) NZ fracture appearance.

Figure 11a–d shows the fracture surface of copper spot joints with and without USV. It exhibits two distinctly different surface features. The horizontal slanted fracture includes several visible defects and reveals a smooth surface. Dimples, void nucleation, growth, and coalescence are not seen on this surface. Additionally, there is no evidence of cleavage facets on the fracture surface. It can be suggested that the failure which happened with the shear planes must have occurred by Orowan’s alternating slip mechanism [19]. The second and third column show that the size of dimples for the HAZ (Figure 11e–h) and the NZ (Figure 11i–l) for samples with USV (2, 4, 6 s) was smaller than the dimples for samples welded without the assistance of USV. The fracture initiated at the NZ, during the coalescence of voids which formed predominantly at the grain boundary (GB). The cleavage facets on the fracture surface are clear. The perpendicular GB to the loading direction was more susceptible to failure than those parallel to the loading direction [20].

### 3.4. Cooling Curves of GTA Spot-Welded Copper Joints

**Figure 12:**
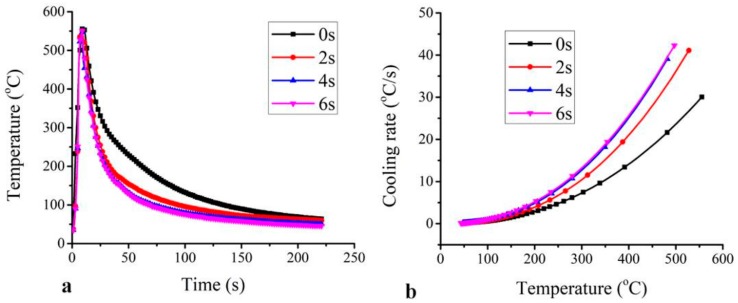
(**a**) The heating and cooling history of welded joints with and without USV; and (**b**) the cooling rate vs. temperature of welded joints.

The temperature histories of the HAZ at various USV times are shown in Figure 12a. It shows that, under the same GTA spot welding, peak temperatures of the HAZ were significantly reduced when rapid cooling by ultrasonic vibration was applied. The peak temperature at the HAZ was as high as 565 °C for 0 s of USV. The duration time above 100 °C of the process assisted with USV (~100 s) was much shorter than that of the process without USV joint (200 s). The cooling rate was evaluated, as shown in Figure 12b. The cooling rate increased with the increase of the USV time. The vibration of the copper sheet with an ultrasonic frequency of 28 KHz increased the heat dissipation rate from the welded joint. The increase of the heat dissipation rate (cooling rate) reduced the chance of grain growth in the HAZ, in addition to a reduction of grain size in the NZ. Finally, the USV supported the formation of equiaxed grains during solidification of the NZ, as shown above in Figure 7d.

### 3.5. The Grain Refining Mechanism of GTA Spot-Welded Copper Joints

**Figure 13:**
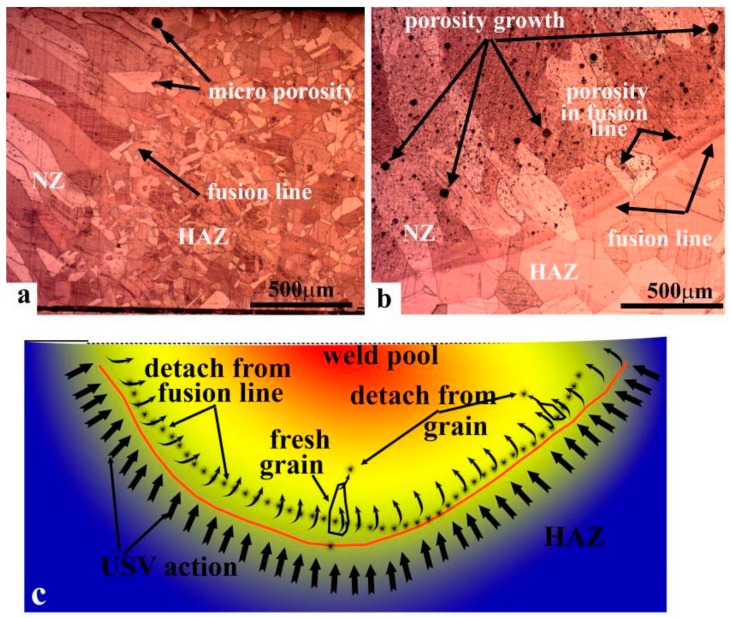
The microstructure around the fusion line for a GTA spot-welded joint (**a**) with USV; (**b**) without USV; and (**c**) the mechanism of the fusion line dissolving and grain refining.

Figure 13a,b illustrate the microstructure and fusion line for welded joints with and without USV, respectively. The width of the semi-melted layer that connects the HAZ and NZ for joints without USV was approximately 100–200 µm. The formation of H_2_O steam bubbles at the fusion line formed a microporosity that may have reduced the electrical conductivity of the copper joint [7]. In this study, the attached transducer at 28 KHz and 60 W provided USV at the contact line between the solid and liquid metal (Figure 13a). The microstructure showed that the shape of the grain had a wide end width for a joint without USV, as shown in Figure 13b. In contrast, the grains in the weld joint with USV consisted of tapered ends and the wide fusion line disappeared (Figure 13a). It can be concluded that the detached particles from the semi-melted fusion line and fragmented new solidified grain moved to form grains inside the NZ under the effect of USV provided through the solid copper to the liquid in the weld pool (Figure 8b,c). The semi-solid particles provided nucleation from the tip of high-density solidified grains; thus, the grain produced a tapered end, as shown in the schematic shown in Figure 13c. Chen et al. [21] investigated the weld pool of a light metal (pure aluminum) subjected to USV. It formed cavitation at the fusion line and refined the grain size in the nugget zone. He concluded that the solidified grain in the NZ, fragmented by high-pressure conditions, was induced during the occurrence of cavitations.

The cavitation action in molten metal was estimated by Eskin [9]. The intensity of acoustics in a melted metal can be expressed by Equation (1):

(1) $$Pa=\sqrt{\frac{2Nac\rho_{0}C_{0}}{S}}$$

where *Pa* is the acoustic intensity; *Nac* is the output power of the ultrasonic device; *ρ*_0_ is the density of molten copper (7.94 g·cm^−3^ [22]), *C*_0_ is the speed of ultrasound in the copper melt (3456 ms^−1^ [22]); and *S* is the area of the ultrasonic field projecting on the liquid metal which is represented by the curvature contact surface of the weld pool with the HAZ, as shown above in Figure 6b. The area of revolution surfaces:

(2) $$S=2\pi \int_{-1}^{1}\sqrt{1+{\left(\frac{dy}{dx}\right)}^{2}dx}$$

The estimated pore pressure was *Pa* = 4.38 MPa; thus, bubbles rose to open at the surface of the molten metal.

### 3.6. The Degassing Mechanism of GTA Spot-Welded Copper Joints

**Figure 14:**
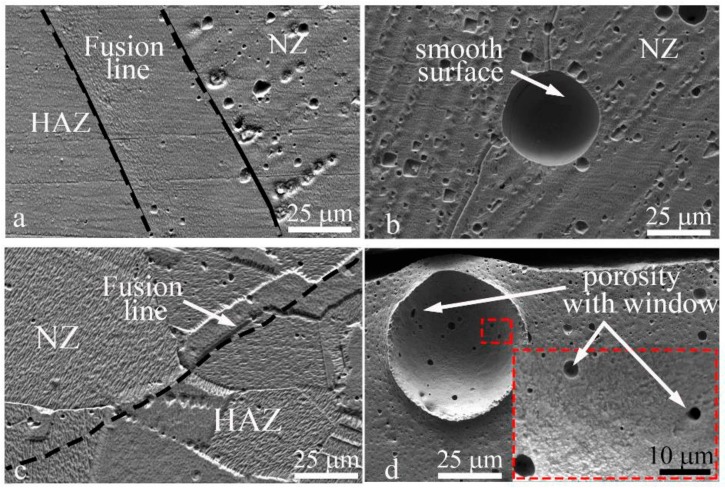
SEM images of porosity at the nugget zone of a GTA spot-welded copper joint made without (0 s USV) (**a**) at the fusion line; (**b**) at the center of NZ; and with 2 s USV; (**c**) at fusion line; and (**d**) at the center of NZ.

Figure 14 illustrates the scanning electron microscope (SEM) images of a copper spot welded joint with and without the assistance of USV. Figure 14a,b show a smooth and spherical surface for interrupted porosity within the fusion line and grains of NZ for the welded joint without the assistance of USV. Figure 14c shows the fusion line of the nugget zone for the copper spot welded joint assisted with USV the fusion line size has been reduced during the effect of USV. Figure 14d shows the porosities that collected through windows to form one enlarged pore in which gas was clustered together from many pores; subsequently, the pore size increased. Pores grow in supersaturated liquid under the diffusion action of gas from the liquid to a bubble, depending on Sievert’s law [23]. The diffusion action and rate depend on the surface energy of the bubble and the pressure in the liquid. When bubbles move to upper parts of the liquid metal, pressure decreases. As the bubble grows, it causes the diffusion of gas atoms from the surrounding liquid in a direction toward the bubble [23]. Porosities combine through windows attributed to USV which provides a vibration to the molten metal and the surface of the pore. An increase in pore size provides an additional opportunity for bubbles to move up through the molten copper, after which they can open at the surface of the nugget.

### 3.7. Electrical Conductivity of GTA Spot-Welded Copper Joints

**Figure 15:**
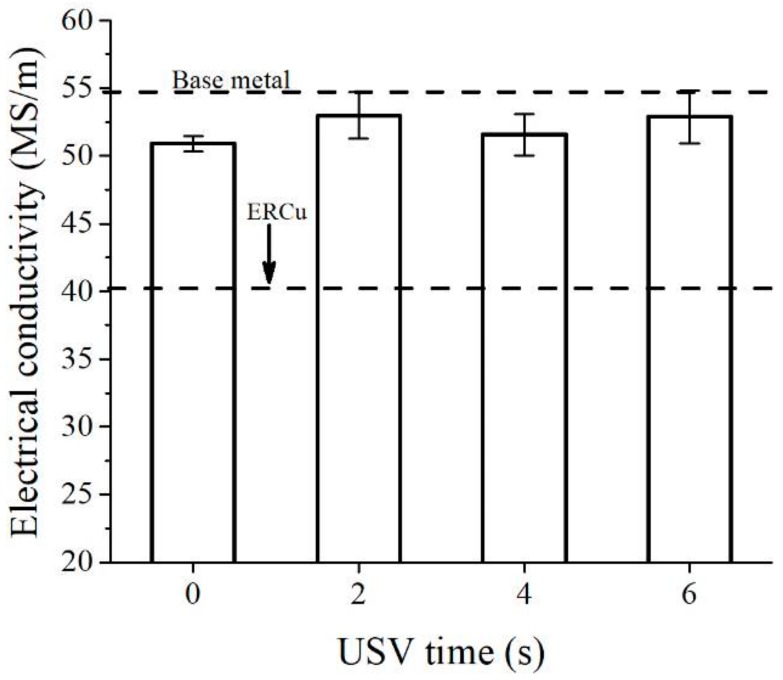
Electrical conductivity of the copper welded joint with and without assistance of USV in comparison with the electrical conductivity of standard welding filler ERCu.

The primary application of pure copper is transference of electrical power. In this study, the electrical conductivity of the copper spot-welded joint was investigated. Figure 15 shows the electrical conductivity of the welded joint without the assistance of USV and the welded joints assisted with USV. Although the process consisted of the protection of the nugget, the electrical conductivity decreased slightly. However, it was still higher than the deposited copper of ERCu [24] or H201 [25] electrodes, which have industrial applications for the welding of copper. In this study, the investigation of the electrical conductivity of copper welded joints assisted with USV was enhanced approximately 3.2 ± 0.2% more than the joint welded without USV assistance. The improvement of electrical conductivity was attributed to the use of a fillerless process and the degassing of porosity away from the nugget zone. Zhang et al. and Kangda Hao et al. [6,25] observed a similar issue when they found the reduction of electrical conductivity (40–70% less than that of the base metal) of a T2 copper joint welded with a hybrid laser-MIG process. They concluded that the impurity elements (alloying elements from the filler metal) and microdefects in the fusion zone were primarily responsible for a decrease in the electrical conductivity of the welded copper joints. Finally, in this study and the above-mentioned studies, the results showed that the coarsened microstructure in the fusion zone and HAZ as well as the possible dislocations formed during the welding process were not the major reasons for the decline of electrical conductivity for welded copper joints. Thus, this research allowed us conduct an in-depth study to produce GTA spot-welded copper joints with ~97.53% electrical conductivity in comparison with the base metal.

### 3.8. Corrosion Behavior of GTA Spot-Welded Copper Joints

**Figure 16:**
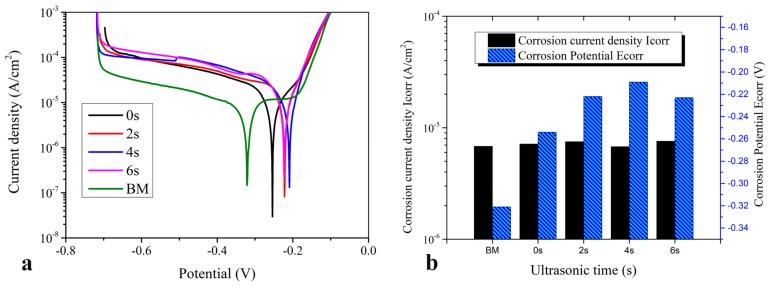
Corrosion resistance of the copper weld joint; (**a**) potentiodynamic polarization curves; and (**b**) corrosion current density and electric potential obtained by Tafel extrapolation.

In Figure 16, both the welds and BM exhibited obvious passivation characteristics during electrochemical corrosion. Corrosion current density (I_corr_) decreased in the sequence of BM, 0 s, 4 s, 6 s, and 2 s, while the corrosion potential (E_corr_) increased in the sequence of BM, 0 s, 6 s, 2 s, and 4 s. Because the smaller I_corr_ denoted a slower corrosion rate and the higher E_corr_ denoted greater difficulty to start the etching, the fresh grains at NZ and HAZ were subjected to ultrasonic peening. Accordingly, the E_corr_ of the weld joints were higher than the BM and the welded joint without ultrasonic peening [26]. Local corrosion was avoided, and the corrosion resistance of welds was improved. The microstructure homogeneity within the weld caused their corrosion resistance to be higher than that of the BM by increasing the electric potential difference of local areas.

## 4. Conclusions

In the present work, the assistance of ultrasonic vibration time (0, 2, 4, and 6 s) for GTA spot-welded copper joints was investigated. The process showed a refining of the grain size at NZ and HAZ simultaneously. Conclusions from the results are as follows:

1. USV had no obvious effect on grain size at NZ. The vibration of NZ for 6 s reduced the grain size ~57%.
2. The cooling rate of the welded metal increased for joints welded with the assistance of USV, which led to an increase of the heat dissipation rate from NZ to the surrounding space. Accordingly, it suppressed the grain growth at HAZ.
3. The refining of grains at HAZ and NZ enhanced the tensile-shear strength of the copper arc spot-welded joints.
4. The mechanism of grain refinement in GTA spot welding of copper assisted with USV was analyzed. It was concluded that USV dissolved the fusion line into small particles that detached to form the nucleation of the new grains at NZ.
5. The mechanism of degassing of NZ was also analyzed. USV activated porosities to be joined and increased internal size and pressure, supporting the movement to be opened at the surface when the pressure of the bubble was 5 MPa.
6. The peening action, degassing, and refining of grains reflected positively on the improvement of electrical conductivity and corrosion behavior of the GTA spot-welded copper joint.
